# Epidermoid Cyst of the Uvula Causing Airway Compromise in a Neonate

**DOI:** 10.7759/cureus.94080

**Published:** 2025-10-07

**Authors:** Dana A Obeid, Amani Obeid, Omar Alghadir

**Affiliations:** 1 Otolaryngology-Head and Neck Surgery, King Saud University Medical City, Riyadh, SAU; 2 Otolaryngology-Head and Neck Surgery, King Fahad Medical City, Riyadh, SAU

**Keywords:** airway obstruction, congenital malformation, epidermoid cyst, neonate, surgical excision, uvular cyst

## Abstract

Congenital uvular cysts are rare lesions in the pediatric population and are typically asymptomatic or discovered incidentally. Only a few cases have been associated with airway obstruction, and none have been reported in a newborn from the Saudi region. This report presents what we believe is the first documented case of a congenital uvular epidermoid cyst in a neonate presenting with airway compromise. We describe a male newborn, delivered at 36 + 5 weeks of gestation, who developed signs of cyanosis and desaturation shortly after birth. During anesthesia for emergent abdominal surgery, a large uvular mass was incidentally identified. Subsequent evaluation revealed a 2 × 7 cm cystic lesion involving the uvula. The patient underwent successful surgical excision of the mass. Histopathological analysis confirmed an epidermoid cyst. Postoperatively, the patient recovered well, with complete resolution of respiratory symptoms. This case underscores the importance of considering uvular cysts in the differential diagnosis of neonatal respiratory distress and highlights the need for awareness among clinicians.

## Introduction

Uvular cysts are a profoundly uncommon entity, especially those of congenital nature across the pediatric and infantile age groups [1,2]. These cysts are mainly of the epidermoid type, a benign, congenital malformation arising during palatal and uvular embryogenic development, which may be associated with other congenital anomalies warranting consideration of genetic counseling. These cysts typically present with mild symptoms or as an incidental finding [3,4]. After initial diagnosis through simple radiological investigations, the mainstay of treatment as undertaken in these cases is complete surgical resection of the cyst as a routine procedure [3,5] with post-op histopathology to confirm the diagnosis. Of the handful of cases reported in the literature, only two comparable cases were found to be presenting with shortness of breath causing airway compromise as emergency cases. However, both patients are of older age and have noticeably smaller cyst sizes as compared to our patient [6,7]. In our search, no cases of uvular cyst causing airway compromise in a newborn were found, and further, no cases of uvular cyst in general were published within our country of Saudi Arabia or rather the whole region. Our study presents the first case of a congenital epidermoid cyst in a newborn with dyspnea and airway obstruction with a substantial uvular mass, as it relates to presentation, workup, management, and subsequent outcome.

## Case presentation

A 31-year-old medically free female, gravida 4, para 2 + 1, came to the labor and delivery unit. Her pregnancy was uncomplicated, and she went into labor at 36 weeks and five days of gestation, with her delivery being spontaneous and no obstetric or medical issues being reported. The newborn baby was a male, weighed 3.1 kg, and had a 35.5 cm head circumference. Apgar scoring was performed as a part of the standard assessment procedure, which was normal, and the baby was sent initially to the postnatal ward. A few hours after being shifted to the postnatal ward, the baby started showing signs of cyanosis and desaturation, with episodes of oxygen desaturation and an abnormally weak cry. The baby was immediately shifted to the neonatal intensive care unit (NICU) for close monitoring.

During the initial physical examination in the NICU, the baby did not show any dysmorphic features or respiratory problems. Initial cardiopulmonary examination showed no acute respiratory problem, heart sounds were regular, and no murmurs were found. Further diagnostic tests were performed due to the persistent episodes of desaturation; on echocardiogram, patent ductus arteriosus (PDA) with a bidirectional shunt was observed. Although the PDA was quantified as small, its impact on hemodynamics was carefully assessed. Additional investigations such as cranial ultrasound showed features of bilateral hydrocephalus. Based on such investigations and findings, the baby was closely monitored under a special team with a special management plan.

**Figure 1:**
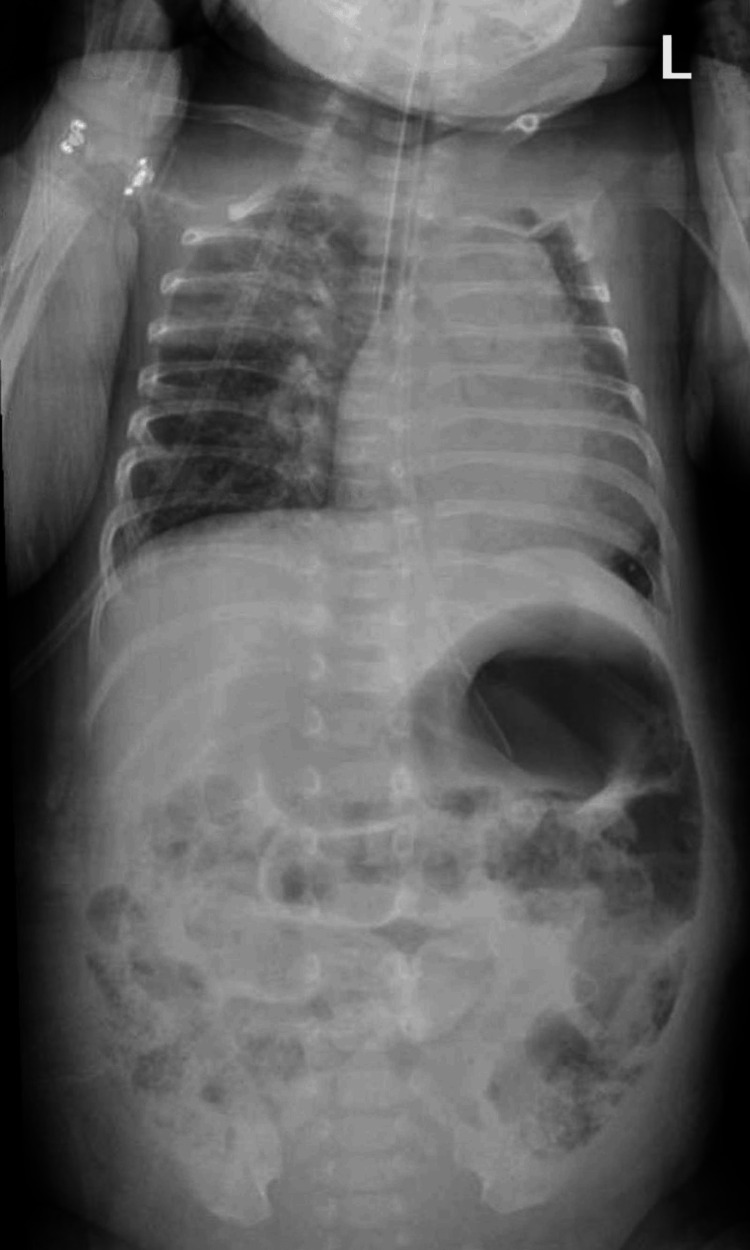
X-ray showing findings consistent with necrotizing enterocolitis (NEC)

**Figure 2:**
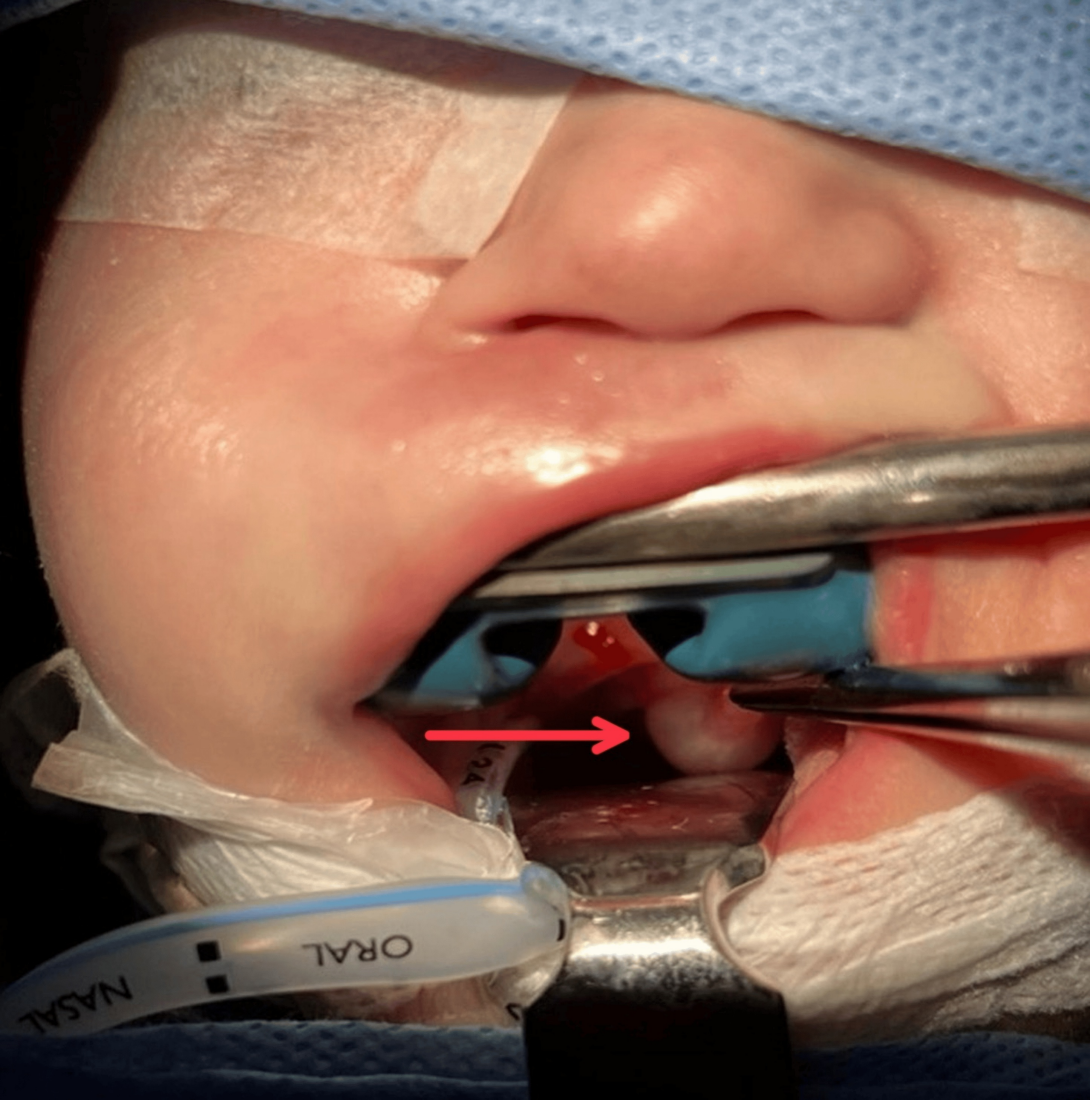
Intraoperative visualization of the large, smooth, fluid-filled cystic mass (arrow) distorting the uvula and soft palate during laryngoscopy for intubation

Thereafter, the baby manifested signs of abdominal distension and feeding intolerance; abdominal X-ray (Figure 1) and ultrasound were performed, which showed the presence of necrotizing enterocolitis (NEC). Concurrently, a perforation was found, which was associated with Meckel's diverticulum, a medical emergency requiring surgical intervention. During anesthesia and intubation, an unexpected mass was observed in the uvula (Figure 2). Concerns were raised by the mass, but given the urgency of the abdominal condition, the focus remained on the NEC surgery, which was completed successfully. After the procedure, the newborn was extubated and transferred to the NICU, and the otolaryngology team was consulted regarding the uvular finding.

**Figure 3:**
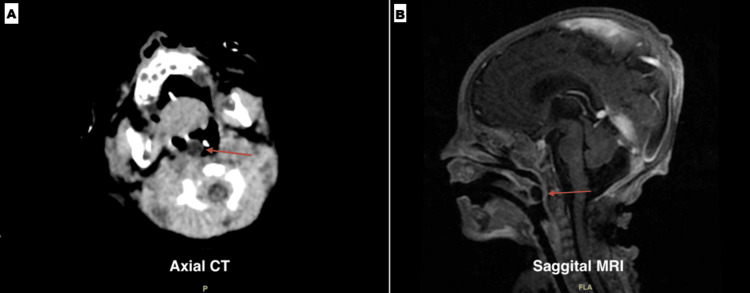
Axial CT (A) and sagittal MRI (B) images demonstrating a well-circumscribed, non-enhancing cystic lesion (arrows) within the uvula. The contents appear homogenous and fluid-filled, consistent with a benign cyst CT: computed tomography; MRI: magnetic resonance imaging

On examination, the team noted a large uvular mass contributing to airway obstruction. Subsequent imaging, including computed tomography (CT) and magnetic resonance imaging (MRI), was obtained to better characterize the lesion (Figure 3). The initial differential diagnosis included Quincke’s edema due to the clinical appearance of uvular swelling; however, imaging demonstrated a well-encapsulated mass rather than transient angioedema. A short course of corticosteroids was given in an attempt to reduce the mass size, but no improvement was observed, and the lesion appeared to gradually enlarge on clinical observation. Consequently, the patient was scheduled for surgical excision once stabilized from the initial surgery.

After the condition of the baby improved from the first surgery, the patient was pushed for surgical excision of the mass. At the time of excision, the mass had increased in size, measuring 2 × 7 cm, and was carefully removed through surgical excision with no complications. During the surgery, the posterior uvular wall was intact, and no evidence was found for involvement of the tissues. The tissue excised during the surgical procedure was sent to the histopathology lab for further examination, which showed benign oral mucosa with a benign squamous cyst (epidermal cyst).

Almost immediately after the surgery, the neonate returned to normal functioning with no further episodes of respiratory distress or desaturation being recorded. The baby was admitted to the NICU postoperatively for a few days for regular observation and was discharged in good health. The family of the neonate was instructed to visit our office regularly to monitor the baby's growth and development, where no further episodes of desaturation or respiratory distress were observed on follow-up. The patient was being followed up with other services for his other issues, and no clear association between them was found.

## Discussion

Cysts in the uvula are infrequent and far in between, with few cases being found across the world. Virtually all uvular cases being reported in literature are of the epidermoid type, particularly of congenital presentation among pediatric and infantile groups. Taking from their name, epidermoid cysts are benign, encapsulated lesions of epidermal-like stratified squamous epithelium that produce and as such are filled with keratin material. These cysts tend to involve any and all parts of the human body including both skin and mucosa layers and even within visceral organs [5,8]. These cysts occur mainly in adult patients and rarely present before puberty, with most cases occurring sporadically, most frequently of traumatic etiology; less commonly, however, epidermoid cysts can be of congenital origin with a possible familial hereditary association [5,8]. Accordingly, cysts in younger patients, in multiple or uncharacteristic locations, should be investigated for Gardner syndrome, Gorlin syndrome, and other possible syndromes associated with such cysts. Direct visualization or through scope, where not feasible, and non-invasive radiology such as CT and X-ray [5,6] are sufficient to identify and manage these cysts, where they show as a heterogeneous, well-encapsulated mass of fat and keratin. Definitive diagnosis is only made through pathological identification; however, since epidermoid cysts are of an indolent, benign nature with very few or no malignant transformation being reported in published research [9,10], histopathology is of less use and is only occasionally performed in more advanced cases or after surgical resection to ensure full resection and lack of neoplastic pathology [5].

The process of development of uvular epidermoid cysts is thought to originate from midline epithelial remnants from the albeit still controversial mechanism of palate formation, more specifically during the process of palatal shelf approximation from opposite sides [4]. Two comprehensive studies on epidermoid cysts and their presenting locations have shown rates of <0.02% and <0.001% involving the oral cavity in general, let alone the less common case of uvular involvement in particular [1,2]. The majority of cases of uvular cysts in the literature are unrelated to our case, with adult presentations reflecting the general epidemiology of these cysts with no congenital association and more related to cocaine and angiotensin-converting enzyme (ACE) inhibitor use [11-13]. Across infantile patients, they typically present with symptoms of snoring and difficulty in feeding or thriving and sometimes are incidentally found [3,14,15]; as such, uvular cysts classically present in older patients and of routine, elective variety. Two similar cases to our study were found, both reporting dyspnea with compromise as a result of an enlarged uvular cyst causing airway blockage; in both cases, the infants were treated successfully by surgical resection with favorable outcomes and full resolution to the symptoms; nevertheless, both cases were past neonatal age, being identified later in life and with much smaller-sized cysts as compared to our case [6,7].

In terms of management, uvular cysts, due to their proximity to the airway, carry a significant risk of compromise, since they are known to get larger, albeit slowly, and have a risk of rupture, inflammation, and/or infection, all of which can interfere and block the airway in a similar mechanism to severe pathologically enlarged tonsils. In the context of this inherent risk, all uvular cases command some form of active surgical intervention to extract the cyst, negating the typical recommendation for observation or laser therapy for small, uncomplicated epidermoid cysts being found elsewhere [5]. The cornerstone and most predominantly performed procedure involves complete surgical excision of the cyst and full removal of its wall lining; the latter of which is of particular importance to prevent keratin reproduction from its epithelial walls and any further recurrence [5,9,10]. Along the same rationale, incision and drainage is highly discouraged across the board for its high risk of recurrence [5,8,10], with only a single instance of a uvular cyst undergoing incision and drainage only as a temporary measure to allow for intubation [16]. Less frequently, and when surgical excision is not feasible, instances of partial and total uvulectomy have been undergone [17,18]. All pediatric cases of all reported ages in the literature displayed no surgical complications from the procedure, and subsequent resolution of their chief, presenting complaints-from the mild cases of snoring and feeding difficulties to the more severe ones of shortness of breath and airway compromise.

## Conclusions

In summary, our case highlights the clinical challenge of managing airway obstruction in a newborn with a small oral cavity and a large uvular mass. The 2 × 7 cm cyst was successfully resected without complication, leading to complete resolution of symptoms. The patient was later discharged in good health, with no anticipated impact on future growth or development. This case underscores the importance of early recognition and careful surgical planning in achieving favorable outcomes.
